# Moyamoya Vasculopathy and Atypical Moyamoya-like Patterns: Insights into Diagnosis and Therapeutic Implications

**DOI:** 10.3390/neurosci7010027

**Published:** 2026-02-15

**Authors:** Rosalinda Calandrelli, Carlo Augusto Mallio, Caterina Bernetti, Luca Massimi, Fabio Pilato

**Affiliations:** 1 Advanced Radiology Center (ARC), Department of Oncological Radiotherapy, and Hematology, Fondazione Policlinico Universitario Agostino Gemelli IRCCS, 00168 Rome, Italy; 2 Research Unit of Diagnostic Imaging and Interventional Radiology, Department of Medicine and Surgery, Università Campus Bio-Medico di Roma, Via Alvaro del Portillo, 21, 00128 Rome, Italy; c.mallio@policlinicocampus.it (C.A.M.);; 3 Fondazione Policlinico Universitario Campus Bio-Medico, Via Alvaro del Portillo, 200, 00128 Roma, Italy; 4 Department of Pediatric Neurosurgery, Fondazione Policlinico Agostino Gemelli IRCCS, Università Cattolica del Sacro Cuore, 00168 Roma, Italy; 5 Department of Neuroscience, Università Cattolica del Sacro Cuore, 00168 Roma, Italy; 6 Department of Medicine and Surgery, Unit of Neurology, Neurophysiology, Neurobiology and Psychiatry, Università Campus Bio-Medico di Roma, Via Alvaro del Portillo, 21, 00128 Roma, Italy

**Keywords:** moyamoya disease, moyamoya syndrome, atypical moyamoya-like patterns, steno-occlusive angiopathy, puff of smoke, imaging

## Abstract

Purpose: The aim of this narrative review is to update current knowledge on Moyamoya vasculopathy (MMV) by addressing key diagnostic debates—including laterality; genetic subtypes; regional epidemiology; and features distinguishing Moyamoya Disease (MMD), Moyamoya Syndrome (MMS) and their mimics. Methods: Key and representative studies were identified through PubMed/MEDLINE and Scopus, focusing on publications from 2014–2025 while also considering earlier seminal works. Results: MMD typically presents with bilateral steno-occlusion of the terminal internal carotid arteries (ICAs) and proximal middle and anterior cerebral arteries (MCAs/ACAs) due to concentric vascular thickening, accompanied by characteristic ‘puff-of-smoke’ collaterals, whereas MMS shows a similar but more often unilateral pattern with fewer collaterals, influenced by the underlying condition. However, this distinction often fails to reflect the full clinical and radiological variability of the Moyamoya spectrum. Atypical moyamoya-like patterns, often confined to M1 or A1 segments, further complicate diagnosis. Clinical manifestations ranged from asymptomatic cases to ischemic or hemorrhagic strokes, and occasionally seizures. Diagnosis relied on multimodal imaging (DSA, MRA, CTA), but genetic mutations, contributing to radiological variability, often complicate differentiation between MMD, MMS, and mimics. Management is pattern-specific: MMS and atypical forms are generally managed conservatively, whereas MMD frequently requires surgical revascularization, particularly in children and symptomatic adults. Nevertheless, variability within diagnostic categories limits the applicability of rigid treatment protocols. Conclusions: Current diagnostic algorithms remain limited. Integrating advanced imaging findings with clinical, genetic, and epidemiological data is essential to define the full disease spectrum, improve diagnostic accuracy, and inform patient management and outcome assessment.

## 1. Introduction

Steno-occlusive diseases involving the terminal portion of the intracranial carotid artery (ICA) and the origin of its major branches represent a rare and heterogeneous group of congenital and acquired conditions—either progressive or non-progressive—that can significantly compromise cerebral blood flow [1,2,3]. Occlusion of the terminal portion of the ICAs, together with the proximal segments of the middle cerebral arteries (MCAs); anterior cerebral arteries (ACAs); and, less commonly, the posterior cerebral arteries (PCAs), accompanied by the development of an abnormal and fragile network of collateral vessels with the characteristic ‘puff of smoke’ appearance, are typical features of moyamoya disease (MMD), traditionally defined as a progressive, idiopathic, and bilateral intracranial arterial stenosis [1,4]. MMD shows notable genetic heterogeneity, with variants in the *RNF213* gene representing the major susceptibility factor—particularly in East Asian populations—and rarer associations with genes such as *ACTA2* and *GUCY1A3* [5,6,7,8,9]. These genetic determinants have been shown to influence age at onset, disease severity, angiographic phenotype, and clinical course, including the risk of ischemic or hemorrhagic events and response to surgical revascularization.

In contrast, when these vascular abnormalities occur in association with other concomitant conditions, the term moyamoya syndrome (MMS) is used, as the underlying disease may contribute to the pathogenesis of the vascular changes [1,3,4]. MMS has been reported in a wide range of disorders including autoimmune or hematological disorders, metabolic diseases, genetic or chromosomal abnormalities, drug toxicity, neoplasms, cranial trauma, and cranial irradiation [10].

However, this distinction often fails to capture the full clinical complexity of the Moyamoya spectrum, as unilateral presentations, atypical patterns, or subclinical forms do not fit neatly within traditional definitions. For this reason, the broader concept of Moyamoya vasculopathy (MMV) has been proposed in recent years as an umbrella entity encompassing MMD, MMS, and atypical variants, offering a more flexible and comprehensive diagnostic framework [11].

Moreover, rare developmental vascular anomalies—such as Aplasia/Twig-like MCA (Ap/T-MCA), arteriovenous malformations (AVM), fibromuscular dysplasia (FMD), and cerebral aneurysms may secondarily induce atypical moyamoya-like changes, thereby mimicking MMV and further complicating the diagnostic process [12,13].

In all these conditions, hemodynamic changes from vascular stenosis, occlusion, or more rarely developmental vascular anomalies—combined with increased mechanical stress on fragile network of intracranial collaterals, may lead to a wide spectrum of clinical manifestations, including headache, seizures, transient ischemic attacks (TIAs), ischemic stroke and hemorrhage [1,14]. Computed tomography angiography (CTA), magnetic resonance angiography (MRA), and digital subtraction angiography (DSA) are reliable tools for identifying and localizing intracranial stenosis, occlusion, or vascular dysplasia, as well as for assessing the collateral circulation network [1,3]. While DSA remains the gold standard for accurately distinguishing MMV from rarer atypical moyamoya-like vascular changes, it does not directly assess parenchymal damage or cerebrovascular reserve (CVR), and may underestimate disease severity [1,15]. Such information is crucial for selecting patients for either conservative or surgical treatment aimed at preventing both ischemic and hemorrhagic stroke. Several studies have described specific features of MMS and MMD [16,17,18,19,20], but only a few have comprehensively reported the full clinical and radiological spectrum of this diagnostically challenging condition [1,3,21,22,23]. Notably, important aspects such as genetic variability, regional epidemiology, and systematic characterization of Moyamoya mimics remain underexplored. Addressing these gaps is essential to improve diagnostic accuracy and patient management.

This narrative review aims to update current knowledge on MMV by addressing key diagnostic debates—including unilateral vs. bilateral forms, genetic subtypes, regional epidemiology, and features distinguishing MMD, MMS and their mimics—to highlight the limitations of traditional classification systems. It also synthesizes emerging evidence to refine diagnosis and support clinically oriented decision-making in both medical and surgical management.

## 2. Material and Methods

### 2.1. Search Strategies

The literature was searched from January 2014 to June 2025 in the medical database PubMed and Scopus using the following MeSH terms: Moyamoya disease, Moyamoya syndrome, Moyamoya vasculopathy, Moyamoya angiopathy, Moyamoya like changes, Twig-like MCA. Seminal older articles were also considered when providing relevant clinical or radiological insights. The combined search ensured broad coverage of the relevant literature, encompassing key aspects of moyamoya vasculopathy, including etiology, pathophysiology, disease laterality, imaging characteristics, clinical presentation, surgical management strategies, outcomes, genetic subtypes, and regional epidemiology.

### 2.2. Overview of Study Selection

The literature review was conducted independently by three authors (a neuroradiologist, a neurologist, and a neurosurgeon), and studies were selected by consensus. In the identification phase, a total of 3463 records were retrieved from PubMed and Scopus. After removing 444 duplicates, 3019 unique records were screened by title and abstract.

During this screening, 2.419 records were excluded because they were not written in English (*n* = 310), were non-original publications such as editorials or conference abstracts (*n* = 561), or were considered not sufficiently representative or relevant to the scope of the narrative review (*n* = 1548).

**Figure 1:**
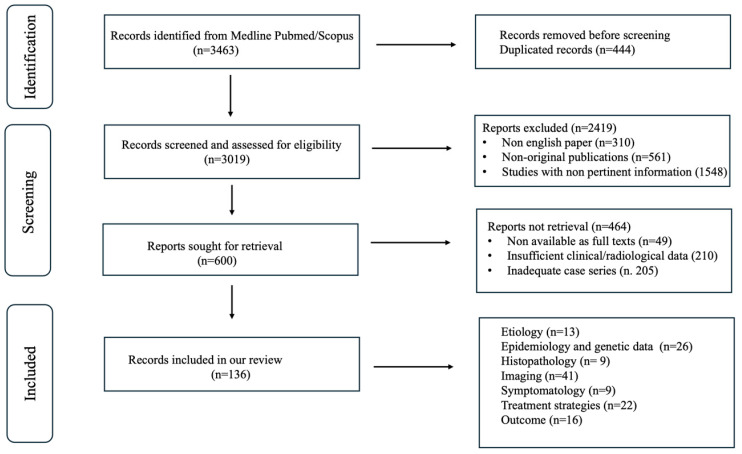
Flow chart of article search and selection.

Subsequently, 600 full-text articles were examined to identify studies providing adequate and pertinent clinical and/or radiological information on moyamoya vasculopathy and its mimics. Of these, 464 were excluded due to insufficient clinical or imaging data (*n* = 210), unavailability of the full text (*n* = 49), or because they did not add substantial value to the narrative discussion (*n* = 205). Ultimately, 136 studies were included. The study selection process is summarized in Figure 1.

## 3. Results

The key results are summarized in Table 1.

## 4. Discussion

### 4.1. Moyamoya Disease

#### 4.1.1. Etiology and Demographics

MMD is a chronic idiopathic cerebrovascular disease characterized by progressive, irreversible steno-occlusive angiopathy, typically affecting the terminal portion of the ICAs and the proximal segments of the MCAs and ACAs [1]. Involvement of the posterior circulation is less common and usually occurs in the advanced stages of the disease [1]. To compensate for inadequate cerebral perfusion, secondary abnormal fine vascular networks—known as ‘moyamoya vessels’—develop. The most common type is the basal moyamoya collateral network, which exhibits the characteristic ‘puff of smoke’ appearance on imaging and primarily consists of extensive lenticulostriate and thalamostriate collaterals, typically oriented perpendicularly to the M1 segment [1,16,20]. Less frequently, collateral pathways may involve dilated anterior choroidal arteries (AchoA), posterior pericallosal arteries, and ethmoidal moyamoya, characterized by enlargement of the ophthalmic, anterior as well as the posterior ethmoidal arteries [1,17]. In advanced stages of the disease, transdural collaterals may also develop, arising from branches of the external carotid artery, as well as from the posterior circulation [1,18].

Bilateral involvement is considered a key diagnostic criterion for MMD [3]. However, longitudinal studies have shown that some patients initially presenting with unilateral disease may progress to bilateral involvement over a follow-up period ranging from 2 to 15 years, suggesting that unilateral MMD (U-MMD) may represent an early disease stage [19,21,29,30,31]. Conversely other authors propose U-MMD as a distinct subtype rather than a mere precursor of bilateral disease [32]. Posterior cerebral artery (PCA) involvement has been reported in only 11.8% of U-MMD patients—significantly lower than in typical MMD [33].

MMD primarily affects individuals of Asian descent, with particularly high prevalence in Japanese and Korean populations [34,35]. Although historically considered rare in Western countries, its incidence has increasingly been reported worldwide in recent years [35]. Reported incidence rates range from 0.34 to 0.94 per 100,000 in Japan and approximately 0.086 per 100,000 in the United States [1]. U-MMD accounts for about 10.5% of all MMD cases in the Japanese population [26]. MMD demonstrates a bimodal age distribution, most commonly affecting children between 3 and 6 years of age and adults between 30 and 40 years [27] and shows a clear female predominance [26].

Genetic susceptibility plays a central role in both disease occurrence and phenotypic variability, contributing to regional differences in clinical presentation. Variants in the RNF213 gene represent the strongest known genetic risk factor for MMD. The *RNF213* p.R4810K variant is strongly associated with early onset, familial aggregation, posterior circulation involvement, and predominantly ischemic presentation, particularly in East Asian populations (Japan, Korea, China) [5,6]. In contrast, rare *RNF213* missense variants affecting conserved residues in the C-terminal E3 ligase domain have been identified in European patients and are frequently associated with more severe or syndromic forms of moyamoya angiopathy, including extracranial vascular involvement and marked intrafamilial phenotypic variability [7,8]. Additional genetic modifiers, such as *GUCY1A3*, may further influence disease severity by affecting inflammation, vascular stability, and endothelial function, potentially through interactions with *RNF213* [9].

Beyond genetic predisposition, non-genetic factors also contribute to disease expression. Secondary insults—including autoimmune mechanisms, infection or inflammation, and prior cranial irradiation—have been implicated in disease onset and progression [3]. Notably, autoimmune diseases appear to be more prevalent in patients with U-MMD than in those with bilateral involvement [26], supporting the role of environmental and immune-mediated factors in modulating disease phenotype.

#### 4.1.2. Histopathology

Histopathological analysis reveals that affected vessels exhibit concentric thickening of the tunica intima, characterized by fibroblast and smooth muscle cell (SMC) proliferation, irregularities of the elastic lamina, thinning of the tunica media, and intraluminal thrombosis—ultimately leading to progressive arterial stenosis and eventual occlusion [3,36]. Overexpression or dysregulation of certain growth factors, including vascular endothelial growth factor (VEGF), basic fibroblast growth factor (bFGF), and hepatocyte growth factor (HGF), is thought to promote intimal hyperplasia and SMC migration [37,38]. Additionally, T-cell and macrophage infiltration within the intima of stenotic vessels, along with IgG deposition in the damaged internal elastic lamina, which facilitates S100A4 protein migration into the intima, further supports the role of immune-mediated mechanisms in vascular narrowing and compensatory collateral vessel formation [38,39].

#### 4.1.3. Clinical Features

**Figure 2:**
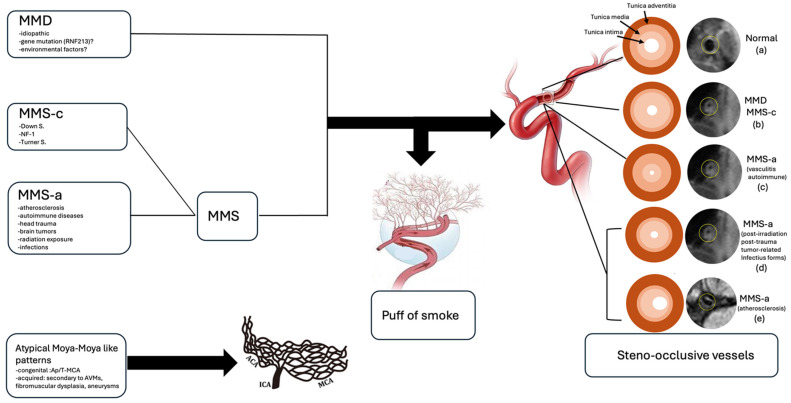
Schematic representation of moyamoya vasculopathy and atypical moyamoya patterns. *Moyamoya disease (MMD) and moyamoya syndrome (MMS)* are characterized by steno-occlusive changes in the terminal ICAs and proximal MCAs/ACAs, with secondary basal collateral networks (“moyamoya vessels,” puff-of-smoke appearance). Normal arterial wall illustrating the three layers: tunica intima (endothelial layer), tunica media (smooth muscle layer), and tunica adventitia (connective tissue layer) (**a**). In MMD and MMS-c, arterial narrowing results from concentric intimal thickening with fibroblast/SMC proliferation, elastic lamina irregularities, and thinning of the media (**b**). MMS-a may arise from: autoimmune/vasculitic forms, with inflammatory hyperplasia of all arterial layers, necrosis, and lymphocytic infiltration (**c**), post-cranial irradiation, post-trauma, tumor-related, or infectious forms, with reactive intimal thickening, medial degeneration, and adventitial fibrosis (**d**); atherosclerotic forms, showing fibrocellular intimal proliferation with foam cells, medial atrophy, and eccentric luminal narrowing (**e**). *Atypical moyamoya-like patterns* may occur in rare congenital vascular anomalies (Ap/T-MCA) or secondary to vascular anomalies (AVM, fibromuscular dysplasia, aneurysm) involving proximal ICA branches, where the MCA is replaced by a plexiform arterial network supplied by ACA, PCA, and hypertrophied choroidal arteries; the terminal ICA is normal. MMS-c, congenital moyamoya disease; MMS-a, acquired moyamoya disease; ICA, internal carotid artery; MCA, Middle Cerebral Artery, ACA, Anterior Cerebral Artery; PCA, Posterior Cerebral Artery; SMC, Smooth Muscle Cell; AVM, Arteriovenous Malformation.

**Figure 3:**
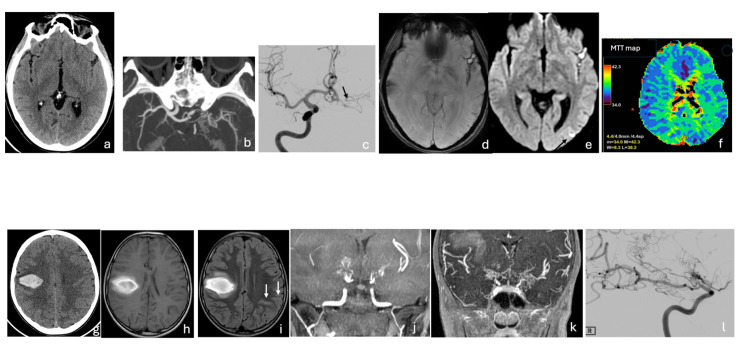
Moyamoya Disease. CT (**a**,**g**), CTA-MIP reconstructions (**b**); DSA (**c**,**l**); MRI: SWI (**d**), DWI (**e**), T1-FSE (**h**), FLAIR (**i**), MRA-TOF (**j**), MRA-CE (**k**), DSC-MTT maps (**f**). (**a**–**f**) A 46-year-old patient with right hemiparesis. Axial CT (**a**) shows obliteration of the left insular subarachnoid spaces. Axial CTA-MIP reconstruction (**b**) shows occlusion of the terminal left ICA and carotid bifurcation, with absence of the M1 trunk of MCA replaced by a plexiform network. Antero-posterior ICA angiogram (**c**) shows normal opacification of the right ICA and its branches and confirm left terminal ICA/M1 occlusion with leptomeningeal collaterals from the ACA (black arrow). MRI reveals hemosiderin in the left Sylvian fissure (**d**) and a small ischemic lesion in temporal lobe (black arrow in (**e**)). Perfusion map demonstrates delayed MCA flow with MTT asymmetry (**f**). (**g**–**l**) A 9-year-old child presenting with sudden loss of consciousness and left hemiparesis. CT (**g**) and MRI (T1 in (**h**), FLAIR in (**i**)) demonstrate a parenchymal hematoma in the corona radiata; linear FLAIR hyperintensities in the left parietal sulci (white arrows in (**i**)) represent prominent leptomeningeal collateral vessels (ivy sign). TOF-MRA-MIP (**j**) and contrast-enhanced MRA (MIP/MPR reconstructions in (**k**)) show bilateral supraclinoid ICA and M1 occlusion with moyamoya vessels from lenticulostriate arteries. Oblique ICA angiogram (**l**) confirms bilateral ICA terminus occlusion (black arrow) and M1 replacement by a plexiform collateral network. CT, Computed Tomography; CTA, Computed Tomography Angiography; MIP, Maximum Intensity Projection; MPR, Multi-Planar Reconstruction; DSA, Digital Subtraction Angiogram; MRI, Magnetic Resonance Imaging; SWI, Susceptibility Weighted Imaging; DWI, Diffusion Weighted Imaging; T1-FSE, T1-Weighted Fast Spin Echo; FLAIR, Fluid Attenuated Inversion Recovery; MRA-TOF, Magnetic Resonance Angiography—Time of Flight; MRA-CE, Magnetic Resonance Angiography—Contrast Enhanced; DSC, Dynamic Susceptibility Contrast; MTT, Mean Transit Time; ICA, Internal Carotid Artery; MCA, Middle Cerebral Artery.

Clinical manifestations vary with age, the severity of stenosis, and the extent of arterial involvement, partly explained by genetic variants and regional epidemiological differences. In children, common presentations include headache, seizures, transient ischemic attacks, and ischemic strokes, often accompanied by progressive cognitive decline. In adults, subarachnoid and intracerebral hemorrhages are more frequent, typically resulting from the rupture of fragile, dilated moyamoya vessels or saccular aneurysms formed secondary to hemodynamic stress [1,28]. Cerebral hemorrhage occurs more often in adults with U-MMD than in those with bilateral MMD [41] (Figure 2 and Figure 3).

### 4.2. Moyamoya Syndrome

#### 4.2.1. Etiology and Demographics

MMS refers to moyamoya disease-like vascular changes occurring in association with underlying congenital or acquired conditions [2]. Congenital disorders, such as Down syndrome, neurofibromatosis type 1 (NF-1), and Turner syndrome, are more common in children, while acquired conditions—like atherosclerosis, autoimmune diseases, head trauma, brain tumors, radiation exposure, and infections—are more frequent in adults [3].

MMS typically involves steno-occlusive changes in the terminal ICA and proximal segments of the MCA and ACA, although isolated involvement of the M2 segment of the MCA has been reported in rare cases [61]. Unilateral involvement occurs more frequently than bilateral disease and is generally associated with less pronounced development of moyamoya collateral vessels [1].

Unlike idiopathic MMD, moyamoya syndrome is not associated with a single defining genetic alteration. However, genetic susceptibility may still play a contributory role in disease development and phenotypic variability. In this regard, variants in the *RNF213* gene—while not considered causative—have been reported as potential susceptibility factors in MMS, potentially facilitating the development of moyamoya-like vascular changes in the presence of secondary insults [3].

Epidemiologically, MMS is more prevalent in Western populations and predominantly affects females, with reported female-to-male ratios ranging from 1.57:1 to 4.25:1 [60]. MMS most commonly presents in adults between 20 and 40 years of age and is more frequently characterized by unilateral involvement and a strong association with underlying conditions such as atherosclerosis, prior cranial irradiation, and genetic syndromes [60].

#### 4.2.2. Histopathology

Histopathologically, congenital MMS shares the occlusion mechanism of MMD [62], while acquired forms show variable mechanisms depending on the underlying cause. Specifically, in autoimmune or vasculitic cases, inflammation induces hyperplasia of the intima, media, and adventitia, along with endothelial inflammation, fibrinoid necrosis, and lymphocytic infiltration, ultimately leading to vessel narrowing and collateral formation [36]. In atherosclerotic forms, vascular occlusion results from intimal fibrocellular thickening due to SMC proliferation and the accumulation of lipid-laden foam cells, accompanied by medial degeneration from muscular atrophy and adventitial thickening caused by chronic inflammation [63]. In cases secondary to cranial irradiation, progressive vascular narrowing results from fibrous intimal thickening with endothelial damage, medial thinning due to degeneration of smooth muscle cells and fibroblasts, and adventitial thickening caused by reactive fibrosis and microvascular injury [23]. In post-traumatic, tumor-related, or infectious forms, vascular stenosis and occlusion result from reactive intimal thickening driven by endothelial hyperplasia, fibroblast activation, and macrophage infiltration, as well as medial degeneration caused by muscle loss or ischemic and inflammatory injury and adventitial thickening due to immune activation, fibrotic hyperplasia, and infiltration of lymphocytes and macrophages [64,65].

#### 4.2.3. Clinical Features

The course of MMS is variable; some cases may remain stable for years, whereas others may progress depending on the underlying cause [66]. Ischemic stroke is the most common clinical manifestation of MMS, while hemorrhagic presentations, including subarachnoid hemorrhage, are less frequent and have been reported in approximately 7% of cases [67].

### 4.3. MMD and MMS- Key Differences and Shared Features

Bilateral involvement is a key diagnostic criteria for MMD, although some patients initially present with unilateral disease that may progress to bilateral involvement over time [3]. Conversely patients with MMS more often present with unilateral steno-occlusive lesions and exhibit less pronounced development of moyamoya collateral vessels [1]. Progression from unilateral to bilateral involvement can also occur in MMS, but the timing and extent are generally less predictable than in MMD [2].

Variants in the *RNF213* gene on chromosome 17 are strongly associated with MMD susceptibility and influence disease phenotype, including early onset and posterior circulation involvement [3,10,22,24,25]. While RNF213 variants may also be detected in some MMS cases, they appear to act primarily as susceptibility alleles rather than causative mutations, contributing to phenotypic variability and sometimes complicating the differential diagnosis [3].

Epidemiologically, MMD predominates in East Asian populations, particularly Japan and Korea [34], whereas MMS is more commonly reported in Western populations and is often associated with underlying congenital or acquired conditions [60]. These differences in geographic distribution, sex ratio, age at onset, and laterality provide additional clues to help distinguish MMD from MMS in clinical practice.

### 4.4. Atypical Moyamoya Patterns

Atypical moyamoya-like patterns can arise from congenital developmental vascular anomalies, such as Ap/T-MCA, or be acquired through vascular remodeling caused by AVMs, FMD, or aneurysms localized at the ICA terminus and its proximal branches (MCA and/or ACA) [13].

#### 4.4.1. Congenital Pattern: Ap/T-MCA

Ap/T-MCA is a vascular anomaly characterized by unilateral absence or hypoplasia of the proximal M1 segment of the MCA, and less commonly of the distal M1 segment or the ACA, without involvement of the ICA terminus or the posterior circulation [12,68,80,82,83,89]. The absent MCA segment is replaced by a plexiform arterial network arising from the ACA, posterior cerebral artery (PCA), and hypertrophied choroidal arteries [13,84,90]. This network demonstrates variable configurations, ranging from complete replacement of the MCA to termination at, or proximal to the MCA bifurcation [91,92]. Lenticulostriate collaterals are generally absent; when present, the plexiform network is typically oriented horizontally, parallel to the M1 axis [68]. Transdural collaterals from external carotid branches are usually lacking, consistent with relatively preserved cerebral perfusion in the affected hemisphere [93].

The etiology of Ap/T-MCA is generally considered congenital, attributed to the embryological persistence of a plexiform network of small vessels [75,76]; however, some investigators propose that Ap/T-MCA may instead represent an acquired abnormality secondary to chronic MCA occlusion, a hypothesis supported by its frequent diagnosis in adulthood and the normal development of the cerebral hemispheres [106]. Although traditionally considered pathogenetically distinct from MMD, genetic evidence has suggested a potential pathogenetic overlap between Ap/T-MCA and moyamoya disease (MMD). Variants of the RNF213 gene, the major susceptibility gene for MMD, have been identified in a subset of patients with Ap/T-MCA, supporting the hypothesis that both conditions may represent phenotypic expressions within a broader spectrum of RNF213-related vasculopathies rather than entirely distinct entities [82,83,84]. However, a definitive association between RNF213 variants and Ap/T-MCA has not yet been established and the genetic contribution remains incompletely understood [84]. Unilateral Ap/T-MCA is the most common presentation, although bilateral cases have also been described [81]. Disease progression is generally absent [13]. The true prevalence of Ap/T-MCA is likely underestimated, as many cases are misclassified under the broader category of moyamoya syndrome (MMS) [80]. Reported prevalence rates range from 0.088% to 1.17%, with a markedly higher frequency observed in East Asian populations, further supporting a potential role for genetic susceptibility [68,84]. Ap/T MCA is often detected incidentally on cross-sectional imaging performed for unrelated indications. Most cases are discovered in adulthood, while pediatric cases remain rare [69,77]. The main clinical manifestation is stroke and approximately 70% of patients with Ap/T-MCA present with hemorrhagic stroke while 20% with ischemic stroke; only a minority remain asymptomatic [70,89,97,98].

#### 4.4.2. Acquired Patterns: Vascular Remodeling Secondary to Vascular Anomalies

AVMs, FMD, and cerebral aneurysms, when located near proximal ICA branches (MCA/ACA), can mimic MMV by causing secondary arterial stenosis or occlusion and triggering the development of characteristic collateral vessels [13,94,95]. These conditions present with a variable age of onset [78,79] and a heterogeneous clinical course, although vascular occlusion is often progressive due to ongoing hemodynamic stress and gradual arterial remodeling [94,95,96]. AVMs occur in approximately 1 in 10,000 people, FMD affects less than 1% of the population, and cerebral aneurysms are present in about 2–5%, with no clear geographic predilection [87,88]. The genetic background of these conditions differs substantially from that of primary moyamoya disease. While a combination of genetic and environmental factors is thought to predispose individuals to FMD and cerebral aneurysms [85], their association with moyamoya-like changes is considered secondary and hemodynamically driven rather than genetically mediated [86]. In contrast no clear gene–environment interaction has been established for AVMs, which are typically sporadic and often congenital in origin [86].

**Figure 4:**
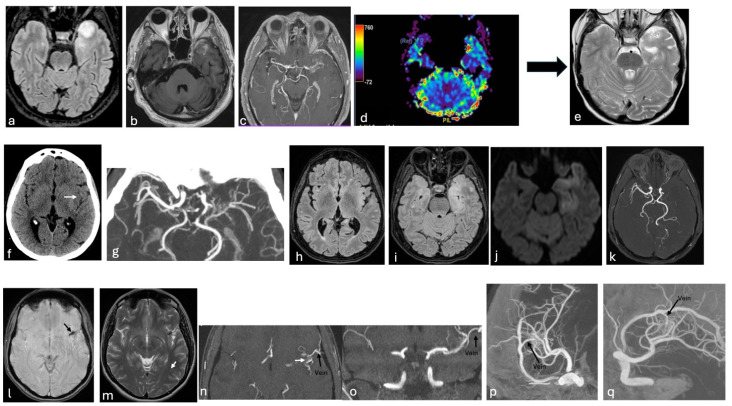
Moyamoya Syndrome and atypical moyamoya-like patterns. CT (**f**), CTA-MIP reconstructions (**g**); DSA-MIP (**p**,**q**); MRI: FLAIR (**a**,**h**,**i**), T2-FSE (**e**,**m**), DWI (**j**), SWI (**l**), T1-FSE- CE (**b**), TOF-MRA (**k**,**n**,**o**), MRA-CE (**c**), DSC-CBV map (**d**). (**a**–**e**) A 45-year-old woman with autoimmune encephalitis presenting with seizures. Brain MRI shows a tumefactive lesion in the left temporal pole (**a**) with patchy post-contrast enhancement (**b**) and increased rCBV on perfusion (**d**), mimicking a tumor. MRA demonstrates multifocal stenosis of the left MCA due to cerebral vasculitis, replaced by a plexiform arterial network (**c**). Six-month follow-up shows gliosis in the temporal pole (**e**). (**f**–**k**) A 52-year-old man with language disturbance and aphasia. CT demonstrates cortico-subcortical hypodensity in the left insula consistent with ischemia (white arrow in (**f**)). CTA-MIP reveals absence of the left M1 segment replaced by a plexiform network, findings compatible with Ap/T-MCA (**g**). FLAIR (**h**,**i**) and DWI (**j**) confirm ischemic changes in the insular, temporal, and parahippocampal regions, and TOF-MRA with 3D reconstruction confirms aplasia of MCA consistent with an Ap/T-MCA anomaly (**k**). (**l**–**q**) A 39-year-old man presenting with daily posterior headaches radiating to the retroauricular region, unresponsive to nonsteroidal anti-inflammatory drugs. SWI brain MRI shows hemosiderin staining (black arrow in (**l**)) in the left Sylvian fissure; no evidence of parenchymal damage on T2-FSE (**m**). TOF-MRA with MIP/MPR (**n**,**o**) and 3D rotational DSA (MIP/MPR from left ICA injection in (**p**,**q**)) reveal irregularity of the left M2 segment and a fine arterial plexus (“puff of smoke”, white arrow in (**n**)) along the short insular gyri, draining into an arterialized vein connecting to the vein of Labbé (black arrows in (**n**,**o**,**p**,**q**)), consistent with a left insular AVM. CT, Computed Tomography; CTA, Computed Tomography Angiography; MIP, Maximum Intensity Projection; MPR, Multi-Planar Reconstruction; DSA, Digital Subtraction Angiogram; MRI, Magnetic Resonance Imaging; SWI, Susceptibility Weighted Imaging; DWI, Diffusion Weighted Imaging; T2-FSE, T2-Weighted Fast Spin Echo; T1-FSE- CE, T1-Weighted Fast Spin Echo-Contrast Enhanced; FLAIR, Fluid Attenuated Inversion Recovery; TOF-MRA, Magnetic Resonance Angiography—Time of Flight; MRA-CE, Magnetic Resonance Angiography—Contrast Enhanced; DSC, Dynamic Susceptibility Contrast; CBV, Cerebral Blood Volume; MCA, Middle Cerebral Artery.

Clinically, these conditions may lead to strokes or TIAs due to impaired cerebral blood flow [99]. AVMs and cerebral aneurysms can also cause hemorrhage upon rupture—AVMs due to the fragility of abnormal vessels, and aneurysms due to weakened arterial walls [86]. Seizures may occur, particularly with AVMs, due to cortical irritation or gliosis [86]. In some cases, aneurysms may compress cranial nerves [100]. FMD, on the other hand, often presents with headaches and pulsatile tinnitus and facial pain [101] (Figure 2 and Figure 4).

### 4.5. Imaging-Guided Differential Diagnosis

Imaging is essential for differentiating typical MMD, MMS, and mimics by assessing vessel stenosis, collateral networks, and parenchymal and hemodynamic changes.

In symptomatic patients CT and CTA are often first-line to evaluate suspected ischemic or hemorrhagic stroke, identify stenotic or occluded vessels, characterize basal moyamoya collaterals, and detect aneurysms or AVMs [1,80]. Despite their utility, CT has limitations as it is less sensitive to early ischemic changes in the hyperacute phase and may underestimate the degree of stenosis in small-caliber vessels. Moreover, exposure to ionizing radiation remains a significant drawback, especially in pediatric patients [107]. MRI and MRA are preferred in children and non-urgent cases, or as complementary second-line modalities following CT/CTA [1]. Diffusion-weighted imaging (DWI) better delineates infarct extent and age-specific ischemic patterns (gyral, borderzone, honeycomb, territorial, multiple-dot, deep lacunar) [1]. T2-weighted gradient-echo and susceptibility-weighted imaging (SWI) are effective for detecting microbleeds and intracranial hemorrhage, providing complementary insight to ischemic stroke assessment and informing prognosis and treatment [108]. Key MRI signs of MMV including leptomeningeal collaterals (“ivy” sign) and prominent deep medullary veins (“brush” sign), are useful indicators of impaired cerebral perfusion [109,110]. The “ivy” sign appears as linear or curvilinear FLAIR hyperintensity or post-contrast T1 enhancement, indicating slow retrograde flow through pial arteries [109]. The “brush” sign, visible on SWI as hypointense medullary veins, reflects an oxygen supply–demand mismatch in hypoperfused tissue [110]. Post-contrast 3D black-blood MRI sequences are useful for diagnosing the underlying cause of vasculopathy by assessing vessel wall morphology and enhancement patterns [111]. MMD typically presents with concentric, non-enhancing steno-occlusive changes associated with vessel wall shrinkage, whereas concentric wall enhancement suggests MMS secondary to vasculitis, and eccentric enhancement is more indicative of MMS related to atherosclerosis [112,113]. MRA imaging—whether 3D time-of-flight or contrast-enhanced techniques—is valuable for identifying the site of stenosis or occlusion and for visualizing collateral networks, which often appear as the classic “puff of smoke” pattern around steno-occlusive lesions. In MMD, these basal collaterals are usually dense and symmetric, reflecting the typical bilateral involvement, whereas MMS tends to show less robust and more asymmetric collateralization, often corresponding to its predominantly unilateral presentation [1,3,16,20]. Acquired moyamoya-like patterns, such as those associated with AVMs, FMD, or aneurysms, usually demonstrate focal or segmental involvement—particularly within the proximal or distal M1 segment—with irregular, localized collateral networks shaped by the underlying vascular lesion rather than by a diffuse arteriopathy [13,94,95].

Advanced perfusion imaging techniques, including CT perfusion (CTP), dynamic susceptibility contrast-enhanced (DSC) MRI, and arterial spin labeling (ASL), enable quantitative assessment of cerebral blood flow, cerebral blood volume, and transit time, facilitating the detection of hemodynamic impairment in the affected hemisphere(s) [51,114,115,116]. In U-MMD perfusion abnormalities typically manifest as localized frontotemporal hypoperfusion, whereas compensatory occipital hyperperfusion may be observed in more advanced stages [117].

Advanced perfusion imaging techniques may also provide a surrogate assessment of cerebrovascular reserve (CVR), as they are effective in characterizing flow delays and hemodynamic dysfunction in moyamoya disease [118]. In particular, DSC-MRI parameters—especially Mean Transit Time—have been shown to correlate with CVR impairment, while ASL allows non-invasive evaluation of cerebral blood flow and arterial transit delays, both before and after revascularization [119,120]. Additionally, functional MRI approaches based on blood-oxygenation-level-dependent (BOLD) signal changes during hypercapnic, breath-hold, or resting-state paradigms allow CVR mapping without ionizing radiation and show good correlation with reference nuclear medicine techniques such as acetazolamide-challenged SPECT or PET, while also being sensitive to post-revascularization changes [121,122,123,124].

DSA remains the gold standard for grading steno-occlusive disease and differentiating idiopathic MMD from MMS or atypical moyamoya [15,51,80]; however, its inability to assess parenchymal function and CVR highlights the added value of multimodal imaging strategies (Table 2).

### 4.6. Severity Score Systems in Moyamoya Disease

Some grading scales are used to support decision-making and grading ischemic risk and they are mainly based on age, hemorrhagic risk and neuroradiological findings.

The Suzuki staging system, based solely on angiography, evaluates the degree of stenosis or occlusion in the terminal ICAs and their major branches, particularly the ACA and MCA, together with the development of collateral vessels [15]. While it has been widely adopted to describe the natural course of disease progression, its limitation lies in the absence of functional or clinical correlation, which restricts its utility for predicting neurological outcomes or surgical risk. The Suzuki grading system for moyamoya disease is divided into six angiographic stages (Table 3).

Steno-occlusive changes in the anterior circulation are typical findings; however, in advanced Suzuki stages, steno-occlusion of the PCA may also develop. PCA involvement is more frequently observed in pediatric-onset MMD and is often associated with a more aggressive disease course, as it reduces leptomeningeal collaterals to the anterior circulation, thereby contributing to ischemic symptoms [40]. To better characterize these changes, Migikura et al. proposed an angiographic staging system specifically for PCA involvement [125] which includes four stages (Table 4).

Recently, Czabanka et al. proposed the Berlin grading system for adult MMD, which integrates morphological and functional MRI/CT data with the Suzuki angiographic staging. This multimodal approach improves clinical severity stratification and enhances the prediction of postoperative neurological morbidity, supporting its adoption in clinical practice [126]. In this system, three independent variables are scored: (1) vessel anatomy, based on DSA findings of stenosis/occlusion and collateral pathways; (2) parenchymal lesions, reflecting ischemic, hemorrhagic, or atrophic changes on MRI; and (3) hemodynamic impairment, assessed by CVR using perfusion imaging combined with a vasodilatory challenge such as acetazolamide administration. The total score ranges from 0 to 6 and classifies disease severity into three grades: Grade I (0–2, mild), Grade II (3–4, moderate), and Grade III (5–6, severe). The detailed scoring system is summarized in Table 5.

Although originally developed for adults, the Berlin grading system has also been applied to pediatric MMD. Nonetheless, neither the Suzuki stage nor the Berlin grading system accounts for clinical severity or individual MMD risk factors.

A novel hemispheric surgical score was developed by an interdisciplinary team to guide surgical decision-making in pediatric MMD, specifically to determine surgical indication and to prioritize which hemisphere should be operated on first. The score is based on clinical symptoms, MRI findings, and DSA, each contributing 0 to 4 points, resulting in a total score ranging from 0 to 12. For patients with a score of 0, medical management and annual follow-up using the ‘moyamoya Protocol’ MRI were recommended. Surgical revascularization was indicated for patients scoring between 1 and 10, with the hemisphere showing the higher preoperative score selected for initial treatment. In contrast, revascularization was not advised for patients with the highest scores (11–12), for whom palliative treatment was deemed appropriate [127] (Table 6).

### 4.7. Treatment

Therapeutic management of Moyamoya vasculopathy is complex, with strategies generally distinguished for idiopathic MMD, secondary MMS and atypical moyamoya-like patterns. Although widely acknowledged in the literature, formal guidelines remain limited and largely observational. The following sections summarize the treatment approaches for each group, emphasizing the key therapeutic differences among them.

#### 4.7.1. MMD Treatment

There is ongoing controversy regarding the treatment of MMD. Since its exact cause remains unknown, various medical therapies—including anticoagulants, antiplatelet agents, and corticosteroids—have been employed, but none have shown clear benefit in preventing disease progression or recurrent cerebrovascular events [21,42,43]. Furthermore, aggressive acute-phase treatments for ischemic stroke such as intravenous infusion of recombinant tissue plasminogen activator (rtPA) or endovascular procedures are not recommended in MMD due to the high risk of hemorrhagic complications and poor outcomes [102,128]. This is largely attributable to the fact that ischemic events in MMD are predominantly driven by chronic hemodynamic impairment rather than thromboembolic mechanisms [14]. Given the limited effectiveness of medical therapy, cerebral revascularization—using a variety of surgical techniques—has long been considered the treatment of choice for patients with ischemic or hemorrhagic symptoms and documented hemodynamic compromise [3,49]. In cases of bihemispheric involvement, surgery is typically performed first on the hemisphere with the greatest impairment of hemodynamic functional status [127] whereas the management of the contralateral asymptomatic side remains controversial. Some authors advocate for prophylactic surgery on the asymptomatic hemisphere, particularly in pediatric patients [117], while others recommend close monitoring and delayed intervention until symptoms or hemodynamic deterioration occur [50].

Although this issue remains controversial, the overarching goal of revascularization is to restore cerebral blood flow, stabilize cerebrovascular hemodynamics, reduce pathological collateral stress, and ultimately prevent future ischemic or hemorrhagic events, thereby improving long-term neurological outcomes and quality of life. Both direct and indirect revascularization techniques, or a combination of the two, may be employed depending on the patient’s condition and surgical expertise [1,22]. Direct anastomotic revascularization includes procedures such as superficial temporal artery (STA)—MCA anastomosis, occipital artery (OA)–MCA anastomosis, and the use of interposition venous grafts. Major indirect revascularization techniques include encephalomyosynangiosis (EMS), which derives its vascular supply from the deep temporal artery, and encephaloduroarteriosynangiosis (EDAS), which utilizes the superficial temporal artery. Variants of these methods—such as encephalomyoarteriosynangiosis (EMAS), encephaloduroarteriomyosynangiosis (EDAMS), and encephalogaleosynangiosis (EGS)—combine different donor arteries and tissues to promote collateral vessel formation [22,44,45]. In cases where posterior circulation is affected, the occipital artery can also be used as a donor vessel in indirect bypass procedures [40]. Indirect revascularization is technically easier to perform, but improvements in cerebral blood flow occur gradually over time [40]. In contrast, direct revascularization provides immediate restoration of blood flow but is more technically demanding and requires a highly skilled surgeon [46,47], increasing the risk of surgical complications such as hyperperfusion syndrome, hemorrhagic stroke, bypass occlusion, anastomotic aneurysm, and scalp necrosis or infection [129,130]. Moreover, direct STA–MCA bypass may be particularly challenging in pediatric patients due to small-caliber vessels and progressive MCA stenosis or occlusion [131].

Multiple surgical series have consistently demonstrated a significant reduction in recurrent ischemic events following revascularization, firmly establishing surgery as an effective treatment strategy for ischemic MMD [132,133,134,135]. Surgical intervention appears to confer greater benefit in children than in adults, as the pediatric form of the disease is typically more aggressive and rapidly progressive [40]. Consequently, conservative management with regular clinical and radiological follow-up may be appropriate for asymptomatic adults without evidence of hemodynamic compromise [48].

The role of revascularization in hemorrhagic MMD has historically been more controversial, owing to concerns that surgical intervention might increase the risk of rebleeding [48]. Nevertheless, several observational studies and surgical series have demonstrated a reduction in recurrent hemorrhage rates following revascularization, particularly after direct bypass procedures, suggesting a protective effect mediated by improved cerebral hemodynamics and a reduction in fragile collateral networks [133,136,137]. More recently, increasing attention has been directed toward the influence of genetic background on disease phenotype and surgical outcomes. In particular, RNF213-associated MMD influences not only age at onset, disease severity, and angiographic features, but also postoperative collateral vessel development and the efficacy of revascularization [138]. Genetic profiling of RNF213 has shown that heterozygosity for the p.Arg4810Lys (p.R4810K) variant was significantly associated with better development of indirect collateral circulation, particularly the deep temporal artery (DTA), whereas homozygosity or the presence of other rare RNF213 variants may correlate with less favorable collateral growth [138].

These findings underscore the emerging role of genetic stratification as a tool to predict surgical outcomes and guide the future personalization of revascularization strategies in Moyamoya vasculopathy.

#### 4.7.2. Treatment of MMS and Atypical Moyamoya-like Patterns

In contrast to MMD, MMS and acquired moyamoya-like patterns are usually managed conservatively at first, because their clinical course depends largely on the underlying disease rather than on a primary moyamoya arteriopathy.

Conservative management is generally recommended for patients with a stable disease course, consisting of regular neuroimaging surveillance and secondary prevention strategies [4,68,70,71]. This approach aims to mitigate the risk of subsequent ischemic events, including strokes and transient ischemic attacks (TIAs), while also allowing for monitoring of disease progression. Cerebral revascularization is typically reserved for patients with progressive neurological symptoms despite optimized medical therapy, as chronic hemodynamic insufficiency may lead to irreversible neurological deficits, developmental delays, or psychomotor disturbances [69,72,73].

However, emerging case-based evidence suggests that in selected patients with atypical moyamoya-like patterns—particularly those with T-MCA anomalies and hemorrhagic presentation—direct STA–MCA bypass may be beneficial in preventing further ischemic or hemorrhagic events and in stabilizing fragile abnormal vascular networks that may contribute to vessel rupture [69,89].

Moreover endovascular intervention remains the first-line approach for treating the primary vascular pathology such as AVM, aneurysm, or fibromuscular dysplasia [91,102].

### 4.8. Outcome Post Treatment

The overall prognosis of MMD is variable and largely depends on clinical presentation, severity of vascular occlusion, hemodynamic status, and timely treatment [40,48,52,53]. Patients managed conservatively face a higher risk of recurrent strokes, TIAs, and disease progression [54]. In contrast, patients undergoing revascularization generally achieve more favorable long-term outcomes, often with partial or complete resolution of ischemic symptoms [55]. Several factors predict poorer postoperative outcomes, including female sex, perioperative hypotension, diabetes, hemodynamic compromise, ischemic MRI lesions, preoperative infarction, fragile collateral networks, and PCA involvement [20,48,56,57,58,59]. Pediatric patients generally respond better to surgical intervention than adults [51] whereas adult patients are at greater risk for postoperative neurological complications [139]. In MMS, prognosis reflects the underlying condition, because treatment often prioritizes etiologic management over bypass surgery [74].

For atypical or overlapping moyamoya-like patterns, prognosis is more heterogeneous and depends on age of onset, stenosis severity, hemodynamic impairment, and the nature of the underlying vascular anomaly. Congenital Ap/T-MCA is usually stable and has a favorable prognosis, although regular monitoring remains necessary [68,80]. Conversely, in acquired moyamoya-like patterns—such as those secondary to AVMs, FMD, or aneurysms—outcomes depends on the risk of ischemia or hemorrhage and on the success of treating the underlying lesion [103,104,105].

## 5. Conclusions

The traditional classification of Moyamoya, which separates idiopathic MMD from secondary MMS, is increasingly insufficient to capture the clinical and radiological variability observed in daily practice, supporting the shift toward a unified framework under the term Moyamoya vasculopathy (MMV). An updated classification that integrates these entities—together with genetic subtyping, regional epidemiological differences, and advanced imaging techniques—is crucial for selecting patients for conservative versus surgical treatment aimed at preventing both ischemic and hemorrhagic events, and for guiding the appropriate timing and intensity of follow-up.

## 6. Future Directions

The increasing recognition that genetic, molecular, and epidemiological mechanisms may overlap across “idiopathic” and “secondary” forms of Moyamoya highlights the potential value of integrated and personalized diagnostic approaches. Such frameworks may enhance diagnostic accuracy, refine ischemic risk stratification, and inform treatment selection—ranging from conservative management in lower-risk patients to surgical or endovascular interventions in those at higher risk. In parallel, advances in genetic and molecular research are opening future avenues for targeted, non-surgical therapies aimed at modulating angiogenesis, inflammation, or vascular remodeling, although these strategies remain experimental. The establishment of large, multicenter prospective registries incorporating integrated clinical, genetic, and imaging data will be essential to refine prognostic stratification and compare long-term outcomes across diverse patient populations.

## Figures and Tables

**Table 1:** Summary of key articles on etiology, epidemiology, histopathology, clinical and imaging findings in Moyamoya vasculopathy and atypical Moyamoya-like patterns.

		Author, Year, [Reference]	Main Findings
MMD	Etiology	Guey et al., 2015 [10]; Kundishora et al., 2021 [24]; Gonzalez et al., 2023 [22]; Ok et al., 2022 [25]; Chen et al., 2016 [26]; Uchiyama et al., 2024 [3]	Idiopathic but genetic (*RNF213* gene mutation) and environmental factors are implicated.
	Age	Chen et al., 2016 [26]; Hayashi et al., 2014 [27].	Bimodal distribution: children (3–6 years), adults (30–40 years). Female predominance
	Side of disease	Uchiyama et al., 2024 [3]; Kim et al., 2016 [28]; Kuroda et al., 2022 [21]; Tian et al., 2022 [29]; Wang et al., 2024 [30]; Strunk et al., 2023 [31]; Houkin et al., 1996 [32]; Mineharu et al., 2024 [33].	Usually bilateral Rarer unilateral MMD (U-MMD)
	Genetic subtypes	Shlobin et al., 2021 [5]; Liao et al., 2017 [6]; Guey et al., 2017 [7]; Santoro et al., 2022 [8], Mineharu et al., 2021 [9]	RNF213 major susceptibility gene (East Asia); rare ACTA2/GUCY1A3 variants; influence age at onset, severity, angiographic pattern, risk of ischemic/hemorrhagic events, and response to surgery
	Regional epidemiology	Kim et al., 2015 [34]; Kainth et al., 2013 [35]	Higher prevalence in East Asia (Japan/Korea)
	Characteristic Imaging findings	Velo et al., 2022 [1]; Liu et al., 2019 [20]; Yamamoto et al., 2019 [16]; Storey et al., 2017 [18]; Fujimura et al., 2019 [17]	Steno-occlusive angiopathy involving mainly terminal ICA, proximal MCA, and proximal ACA; posterior circulation involvement is rare. Moyamoya collateral networks (puff of smoke appearance) typically form perpendicular to the M1 segment via lenticulostriate and thalamo-striate vessels; transdural collaterals develop in advanced stages of the disease.
	Histopathology of stenosis	Uchiyama et al., 2024 [3]; Fox et al., 2021 [36]; Shirozu et al., 2023 [37]; Xu et al., 2024 [38]; Fujimura et al., 2018 [39].	Tunica intima: concentric thickening Tunica media: thinning Tunica adventitia: normal
	Disease course	Karsonovich et al., 2025 [40]	Progressive, more commonly in children
	Clinical manifestations	Velo et al., 2021 [1]; Kim et al., 2016 [28]; Yu et al., 2019 [41]	Childhood: Headache, seizures, transient ischemic attacks, ischemic strokes Adults: Subarachnoid and intracerebral hemorrhages
	Therapy	Uchiyama et al., 2024 [3]; Gonzalez et al., 2023 [22]; Karsonovich et al., 2025 [40]; Yamada et al., 2016 [42]; Liu et al., 2023 [43]; Kuroda et al., 2022 [21]; Acker et al., 2018 [44]; Qian et al., 2015 [45]; Porras et al., 2018 [46]; Wang et al., 2017 [47]; Kim et al., 2021 [48], Uchiyama et al., 2020 [49]; Nagata et al., 2006 [50]	Children: Direct (STA-MCA; OA-MCA) and indirect (EMS, EDAS) anastomotic revascularization Adults: Conservative treatment if asymptomatic and without parenchymal hemodynamic impairment; surgical treatment in symptomatic and in cases of impairment of hemodynamic functional status.
	Outcome	Karsonovich et al., 2025 [40]; Xie et al., 2022 [51]; Kim et al., 2021 [48]; Alnaqeeb et al., 2025 [52]; Ladner et al., 2017 [53]; Hayashi et al. 2023 [54]; Karki et al. 2024 [55]; Funaki et al. 2015 [56]; Jeon et al. 2018 [57]; Im et al. 2021 [58]; Lin et al. 2019 [59]	Surgical revascularization procedures are associated with more favorable long-term outcomes, especially in pediatric patients
MMS	Etiology	Uchiyama et al. 2024 [3]; Phi et al., 2015 [2]	*MMS-c.* Congenital disorders in children: Down syndrome, NF-1, and Turner syndrome *MMS-a.* Acquired conditions in adults: atherosclerosis, autoimmune diseases, head trauma, brain tumors, radiation exposure, and infections
	Age	Acker et al. 2016 [60]	20–40 years; female predominance
	Side of disease	Hayashi et al. 2014 [27]	More commonly unilateral
	Genetic subtypes	Uchiyama et al., 2024 [3]	No single causative gene; *RNF213* variants may confer increased susceptibility
	Regional epidemiology	Acker et al. 2016 [60]	More prevalent in Western countries
	Characteristic Imaging findings	Velo et al., 2021 [1]; Hayashi et al., 2014 [27]; Jimenez et al., 2016 [61].	Steno-occlusive angiopathy involving terminal ICA, proximal MCA, and proximal ACA; rare stenoses of M2 segment of MCA may occur. Moyamoya collateral networks with “puff of smoke” appearance develop (less pronounced than MMD)
	Histopathology of stenosis	Lin et al., 2012 [62]; Fox et al., 2021 [36]; Jiang et al., 2013 [63]; He et al., 2025 [23]; Sharfstein et al., 2007 [64]; Czartoski et al., 2006 [65]	*MMS-c:* concentric thickening of tunica intima, thinning of tunica media and normal tunica adventitia MMS-a (autoimmune or vasculitic cases): hyperplasia of tunicae intima, media, and adventitia. MMS-a (atherosclerotic forms, cranial irradiation, post-traumatic, tumor-related, or infectious forms): thickening of tunica intima, thinning of tunica media and thickening of tunica adventitia
	Disease course	Phi et al., 2016 [66]	Usually non-progressive
	Clinical manifestations	Bejot et al., 2017 [67]	Ischemic stroke and intracranial hemorrhage
	Therapy	Ribigan et al., 2020 [4]; Onoue et al., 2021 [68]; Tsukada et al., 2024 [69]; Tashiro et al., 2016 [70]; Jeong et al., 2022 [71]; Inoue et al., 2016 [72]; Matsunaga et al., 2018 [73]	Conservative management: neuroimaging surveillance and secondary prevention strategies directed at treating the underlying pathology Anastomotic revascularization in cases of progressive neurological symptoms and chronic hemodynamic insufficiency.
	Outcome	Das et al. 2021 [74]	It depends on early diagnosis and the timely initiation of appropriate treatment.
Atypical moyamoya- like patterns	Etiology	Cho et al., 2019 [75]; Akkan et al., 2015 [76]	-Ap/T-MCA: congenital developmental anomaly of MCA
		Uchiyama et al., 2017 [13]	-Acquired forms: vascular adaptation secondary to AVM, FMD, or aneurysm
	Age	Tsukada et al., 2024 [69]; Takeda et al., 2022 [77]	-Ap/T-MCA: childhood or young adulthood
		Pasquini et al., 2015 [78]; Stanishevskiy et al., 2024 [79]	-Acquired forms: variable according to underlying pathology
	Side of disease	Goto et al., 2022 [80]; Shirokane et al., 2020 [81]	Unilateral; rare bilateral cases
	Genetic subtypes	Ota et al., 2021 [82]; Inoue et al., 2022 [83]; Nakajima et al., 2024 [84]	-Ap/T-MCA: Possible association with RNF213 variant; no definitive causal relationship established.
		Van der Niepen et al.,2021 [85]; Hofmeister at al., 2000 [86]	-Acquired forms: No moyamoya-specific genetic signature identified
	Regional epidemiology	Onoue et al., 2021 [68]; Nakajima et al., 2024 [84]	-Ap/T-MCA: Rare and likely underdiagnosed; higher prevalence in East Asian populations.
		Olin et al., 2014 [87]; Rinkel et al., 1998 [88]	-Acquired forms: no geographic predilection.
	Prevalence	Onoue et al., 2021 [68]; Nakajima et al., 2024 [84]	-Ap/T-MCA: rare (0.09–1.2%), more common in East Asians.
		Olin et al., 2014 [87]; Rinkel et al., 1998 [88]	-Acquired forms: 1–5% (variable depending on the underlying disease).
	Characteristic Imaging findings	Zedde et al., 2024 [12]; Uchiyama et al., 2017 [13]; Onoue et al., 2021 [68]; Goto et al., 2022 [80]; Ota et al., 2021 [82]; Inoue et al., 2022 [83]; Takarada et al., 2021 [89]; Lutz et al., 2018 [90]; Nakajima et al., 2024 [84]; Goto et al., 2019 [91]; Chetoui et al., 2024 [92]; Seo et al., 2012 [93].	-Ap/T-MCA: aplastic or absent MCA segment replaced by a plexiform arterial network. Transdural collaterals are lacking.
		Uchiyama et al., 2017 [13]; Ahn et al., 2010 [94]; Ashleigh et al., 1992 [95]	-Acquired forms: arterial stenosis or occlusion (usually of the MCA) secondary to vascular anomaly and development of characteristic collateral vessels.
	Disease course	Uchiyama et al., 2017 [13]	-Ap/T-MCA: non-progressive/stable
		Ahn et al., 2010 [94]; Ashleigh et al., 1992 [95]; Noh et al., 2014 [96]	-Acquired forms: variable course, usually progressive
	Clinical manifestations	Takarada et al., 2021 [89]; Viso et al., 2021 [97]; Tashiro et al., 2016 [70]; Lang et al., 2017 [98]	-Ap/T-MCA: about 10% asymptomatic; majority (~90%) symptomatic, often with hemorrhagic or ischemic stroke presentations.
		Gupta et al., 2014 [99]; Hofmeister et al., 2000 [86]; Almaghrabi et al., 2021 [100]; Bagh et al., 2018 [101]	-Acquired forms: variable clinical presentation including seizures, hemorrhagic or ischemic stroke, focal deficits, or headaches; symptoms depend on underlying AVM, FMD, or aneurysm.
	Therapy	Onoue et al., 2021 [68]; Tashiro et al., 2016 [70]	-Ap/T-MCA: Conservative management if asymptomatic; medical therapy (antiplatelets, stroke prevention) if symptomatic.
		Zhang et al., 2015 [102]	-Acquired forms: Treatment tailored to underlying pathology. AVM (surgery, embolization, radiosurgery); FMD (medical therapy, angioplasty); aneurysms (clipping, coiling).
	Outcome	Onoue et al., 2021 [68]; Goto et al., 2022 [80]; Nurimanov et al., 2023 [103]; Yu et al., 2016 [104]; Kesav et al., 2023 [105]	Ap/T-MCA: favorable prognosis Acquired forms: It depends on the risk of ischemic or hemorrhagic events, influenced by the nature and progression of the underlying vascular anomaly

RNF213, Ring Finger Protein 213; MMS, Moyamoya Syndrome, MMD, Moyamoya Disease; U-MMD, Unilateral Moyamoya Disease; ICA, Internal Carotid Artery; *MCA*, Middle Cerebral Artery; ACA, Anterior Cerebral Artery; STA-MCA, Superficial Temporal Artery-Middle Cerebral Artery; *OA-MCA*, Occipital Artery–Middle Cerebral Artery; EMS, Encephalomyosynangiosis; EDAS, Encephaloduroarteriosynangiosis; MMS-c, Moyamoya Syndrome-congenital; MMS-a, Moyamoya Syndrome-acquired; NF-1, Neurofibromatosis type 1; Ap/T-MCA, Aplasia/Twig-like Middle Cerebral Artery; AVM, Arteriovenous Malformation; FMD, Fibromuscular Dysplasia.

**Table 2:** Key differential imaging features of moyamoya disease, moyamoya syndrome, and acquired moyamoya-like patterns.

	MMD	MMS	*Acquired Patterns*
Distribution of steno-occlusion	Terminal ICA; proximal MCA/ACA; PCA in advanced stages	Terminal ICA; proximal MCA/ACA/PCA; distribution influenced by underlying disease	Segmental M1 involvement (proximal or distal); localized to the site of primary lesion
Laterality	Typically bilateral	Typically unilateral	Unilateral, lesion-dependent
Collateral pattern	Dense, symmetric basal collaterals (“puff-of-smoke”)	Less robust, often asymmetric basal collaterals (“puff-of-smoke”)	Collaterals focal/irregular; related to AVM feeders, FMD loops, or aneurysm-related flow changes
Progression	Predictable bilateral progression	Less predictable bilateral progression	Generally absent
Vessel wall imaging	Concentric, non-enhancing stenosis	Concentric or eccentric enhancing wall thickening	Variable: Eccentric enhancement (aneurysm/dissection); segmental irregularities (FMD); flow-related changes (AVM)
Parenchymal findings	Chronic ischemia; watershed infarcts; FLAIR “ivy sign”	Variable ischemic changes depending on etiology; asymmetric ivy sign	Findings specific to the primary pathology: AVM-nidus/hemorrhage-related; FMD-dissection related infarcts; aneurysm- SAH-related
Perfusion profile	Bilateral impaired cerebrovascular reserve	Unilateral/asymmetric reduction	Focal hypoperfusion or hyperperfusion near AVM

MMD = Moyamoya disease; MMS = Moyamoya syndrome; AVM = arteriovenous malformation; FMD = fibromuscular dysplasia; ICA = internal carotid artery; MCA = middle cerebral artery; ACA = anterior cerebral artery; PCA = posterior cerebral artery; M1 = first segment of the middle cerebral artery; SAH = subarachnoid hemorrhage; FLAIR = Fluid-Attenuated Inversion Recovery.

**Table 3:** Angiographic Suzuki grading system of moyamoya disease.

Stages	Findings
Stage I	Narrowing of the terminal portion of the ICA.
Stage II	Initiation of abnormal collateral (“moyamoya”) vessel formation at the base of the brain with dilation of the intracerebral main arteries
Stage III	Intensification of moyamoya vessels with further ICA and intracerebral main arteries stenosis.
Stage IV	Reduction in moyamoya vessels; advanced ICA, ACA, MCA steno-occlusion with development of external-to-internal carotid system collaterals (e.g., via the external carotid artery).
Stage V	Further diminution of moyamoya vessels; extracranial collaterals become more dominant from the external carotid artery.
Stage VI	Disappearance of moyamoya vessels; with cerebral circulation maintained only by the external carotid artery or the vertebral artery

ICA, Internal Carotid Artery; *MCA*, Middle Cerebral Artery; ACA, Anterior Cerebral Artery.

**Table 4:** Angiographic Posterior Cerebral Artery Staging of Moya-Moya Disease.

Stages	
Stage I	No occlusive changes in the PCA
Stage II	Stenosis in the PCA with or without slightly developed PCA moyamoya vessels
Stage III	Severe stenosis or virtually complete occlusion of the PCA with well-developed PCA Moyamoya vessels
Stage IV	Occlusion of the PCA with decreased PCA Moyamoya vessels

PCA, Posterior Cerebral Artery.

**Table 5:** Summary of Berlin Grading System.

Variables	Characteristics	Points
Vessel Anatomy (DSA)	Stenotic or occlusive lesions with typical moyamoya vessels, but without intracranial or extra-intracranial collateral pathways.	1
	Stenosis/occlusion with moyamoya vessels and additional intracranial collaterals, such as leptomeningeal and/or pericallosal anastomoses.	2
	Stenotic or occlusive lesions accompanied by the presence of extra-intracranial collaterals.	3
Parenchymal Lesions (MRI)	No signs of ischemia, hemorrhage, or atrophy	0
	Evidence of cerebral infarction, intracerebral hemorrhage, or cerebral atrophy.	1
Hemodynamic Impairment (PWI, CTP)	Normal perfusion with preserved CVR.	0
	Impaired CVR without critical perfusion deficit.	1
	Severely reduced CVR, indicating critical hemodynamic compromise.	2

DSA, Digital Subtraction Angiography; MRI, Magnetic Resonance Imaging; PWI, Perfusion-Weighted Imaging; CTP, CT Perfusion; CVR, Cerebrovascular Reserve.

**Table 6:** Novel hemispheric surgical score.

Variables for Each Hemisphere	Severity	Points
Clinical Events	Asymptomatic	0
	One TIA with reversible neurological deficit. Seizure	1
	More than 1 TIA	2
	One ischemic Arterial Stroke	3
	More than one Ischemic Arterial Stroke or severe neurological deterioration (spastic/dystonic quadriparesis, severe symptomatic developmental delay)	4
MRI Findings	Normal or small white matter lesions with normal MTT/CBF	0
	Small white matter lesions with normal CBF but prolonged MTT	1
	Low CBF and/or infarcts in watershed areas	2
	Territorial infarcts	3
	Cortical atrophy outside the infarct area	4
DSA Findings	Stenosis with leptomeningeal collaterals and/or from the ECA, without contralateral involvement	0
	Stenosis with leptomeningeal collaterals and/or from the ECA, with contralateral involvement	1
	Stenosis without collaterals (leptomeningeal and/or from the ECA), without contralateral involvement	2
	Stenosis without collaterals (leptomeningeal and/or from the ECA), with contralateral involvement	3
	Involvement of the Posterior Circulation	4

Magnetic Resonance Imaging (MRI); Digital Subtraction Angiography (DSA); TIA, Transient Ischemic Attack; MTT, Mean Transit Time; CBF, Cerebral Blood Flow; ECA, External Carotid Artery. *Total Score per Hemisphere*: 0 = Medical treatment and annual follow-up (both clinical and with non-invasive neuroimaging, 1–10 = Indirect revascularization. If there is bihemispheric involvement: start with the hemisphere with the highest score. 11–12 = Palliative treatment.

## Data Availability

No new data were created or analyzed in this study.
